# Case report: A follow-up report of omental packing and drug therapy for canine prostate adenocarcinoma

**DOI:** 10.3389/fvets.2024.1444684

**Published:** 2024-11-12

**Authors:** Yanan Li, Dapeng Li, Tianwen Ma, Chengwei Wei

**Affiliations:** ^1^Heilongjiang Key Laboratory of Animal Disease Pathogenesis and Comparative Medicine, College of Veterinary Medicine, Northeast Agricultural University, Harbin, China; ^2^Zhejiang Baoyuan Animal Husbandry Co., Ltd., Hangzhou, China; ^3^Dr. Pet Animal Hospital Central Hospital, Shenyang, China; ^4^Animal Clinical Teaching Hospital, Northeast Agricultural University, Harbin, China

**Keywords:** omental packing, prostatic cyst, prostate adenocarcinoma, canine, drug therapy

## Abstract

Canine prostate is susceptible to diseases such as cysts, abscesses, and tumors. A 15-year-old male castrated Chinese rural dog underwent staged treatment. Preliminary diagnosis is based on examination results, including clinical symptoms (tenesmus, dysuria, frequent urination, and hematuria); hematology (elevated neutrophil count); X-rays (swelling of the prostate); ultrasound examination (less uniform echo in the prostate region, no echo effect in parenchyma); biopsy smear of prostate tissue (large number of neutrophils and rod-shaped bacteria). Therefore, the dog was preliminarily diagnosed with a prostate abscess. Antibiotic therapy was used for treatment. Three days later, the symptoms of hematuria and frequent urination did not improve, and the state was poor. The owner was advised to undergo surgical treatment-omental packing. Meanwhile, bacterial culture identification, drug sensitivity test and histopathological examination were performed. Pathological diagnosis was prostate adenocarcinoma. Subsequently, antibiotic therapy with enrofloxacin and antineoplastic maintenance therapy with mitoxantrone were administered. Six months later, the dogs were followed up, and the results showed no disease in the prostate tissue and no metastatic lesions. This is the report describing the use of omental packing for the treatment of prostate adenocarcinoma in dogs. In order to provide an important theoretical basis for the treatment of prostate cancer - omental packing into veterinary routine.

## 1 Introduction

Canine prostate may suffer from various diseases, the most common of which are benign prostatic hyperplasia, prostatitis, prostatic abscess, prostatic cyst, and prostatic tumor (1). Among them, prostatic abscesses are often considered to be sequelae of prostatitis or may be associated with cystic hyperplasia (2). Prostate tumors are rare, with an incidence of 1.1 to 5.3% in dogs with prostate disease (1, 2). Due to the lack of specific antigen (PSA) markers and effective screening methods for canine prostate cancer, early diagnosis is difficult. Histopathologic examination, though, is the gold standard for diagnosis, but it is a kind of invasive operation, will produce a considerable cost and risk (3). Therefore, the host will not accept it.

Antibiotic therapy was often used for canine prostate disease but was abandoned due to poor efficacy and used only as an adjuvant therapy. Surgical treatment of canine prostatic abscesses may involve drainage, excision, or bagging (4). Canine prostate cancer is divided into prostate adenocarcinoma (AC) and prostate urothelial carcinoma (UC), which are aggressive and metastatic (5) (the probability of metastasis is as high as 70–80%, and it is mainly concentrated in tissues such as lymph nodes, lungs, and bones). The prognosis is often poor, with untreated animals surviving only about 10 days to 1 month. There is no good strategy for the treatment of prostate cancer, and the common treatment measures include medical therapy, radiotherapy and surgery (6). The current surgical treatment for prostate cancer is a total prostatectomy with a pubotomy followed by chemotherapy. However, this method has many disadvantages, such as large tissue destruction, long operation time, and postoperative complications such as limited hind limb movement and urinary incontinence, which have a great impact on the recovery of elderly dogs (7). Common drug therapies include nonsteroidal anti-inflammatory drugs and chemotherapy drugs. The median survival time of drug-treated canines was 6.9 months, while that of untreated canines was 0.7 months, which was a significant difference (8). There is a need for a method that can have a relatively small impact on dogs (especially elderly dogs) to treat canine prostate disease. The greater omentum has rich blood circulation, strong regeneration ability, and absorptive function. Omental packing is a method to treat chronic infections using omental tissue. It is easy to attach to the surrounding tissues to form a wide range of collateral circulation, which is often used in the treatment of infectious diseases such as prostate abscess and empyema. Therefore, Omental packing has the advantages of less tissue destruction, short operation time and fewer complications, which is conducive to postoperative recovery of dogs, especially elderly dogs.

Therefore, the early diagnosis of canine prostate cancer as well as the development of an informed treatment plan are essential. To better understand the course of canine prostate cancer, this study reports an uncommon but highly effective therapy, omental packing. This contribution aims to strengthen the scientific community in the development of therapies for canine prostate cancer.

## 2 Case description

### 2.1 Patient clinical examination

A 15-year-old Chinese rural dog weighing 10.75 kg came to our hospital. The main clinical symptoms observed included tenesmus, dysuria, frequent urination, and hematuria. The owner described that the dog had been ill for more than half a year, and recently showed symptoms of decreased appetite. Initial blood routine, biochemical, X-ray, ultrasound, and tissue smear examinations were required.

### 2.2 Abdominal ultrasound and X-ray

**Figure 1:**
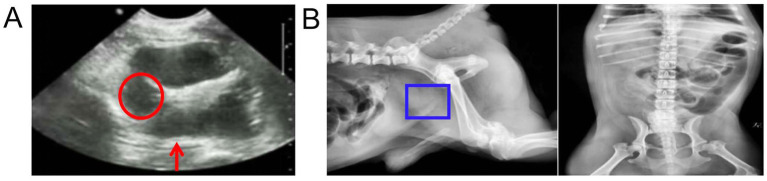
Ultrasound and X-ray examination of the prostate. (A) Ultrasound images. The red circle represents no echo in the parenchyma and the red arrow represents the echo was enhanced in the surrounding tissue. (B) X-ray image. The blue boxes represents swelling of the prostate region.

Ultrasound results showed that the prostate capsule was smooth, the outline was clear, the internal echo was not uniform, there was no echo in the parenchyma, and the echo was enhanced in the surrounding tissue (Figure 1A). X-ray findings revealed swelling of the prostate region (Figure 1B).

### 2.3 Blood and urine tests and cytology

**Figure 2:**
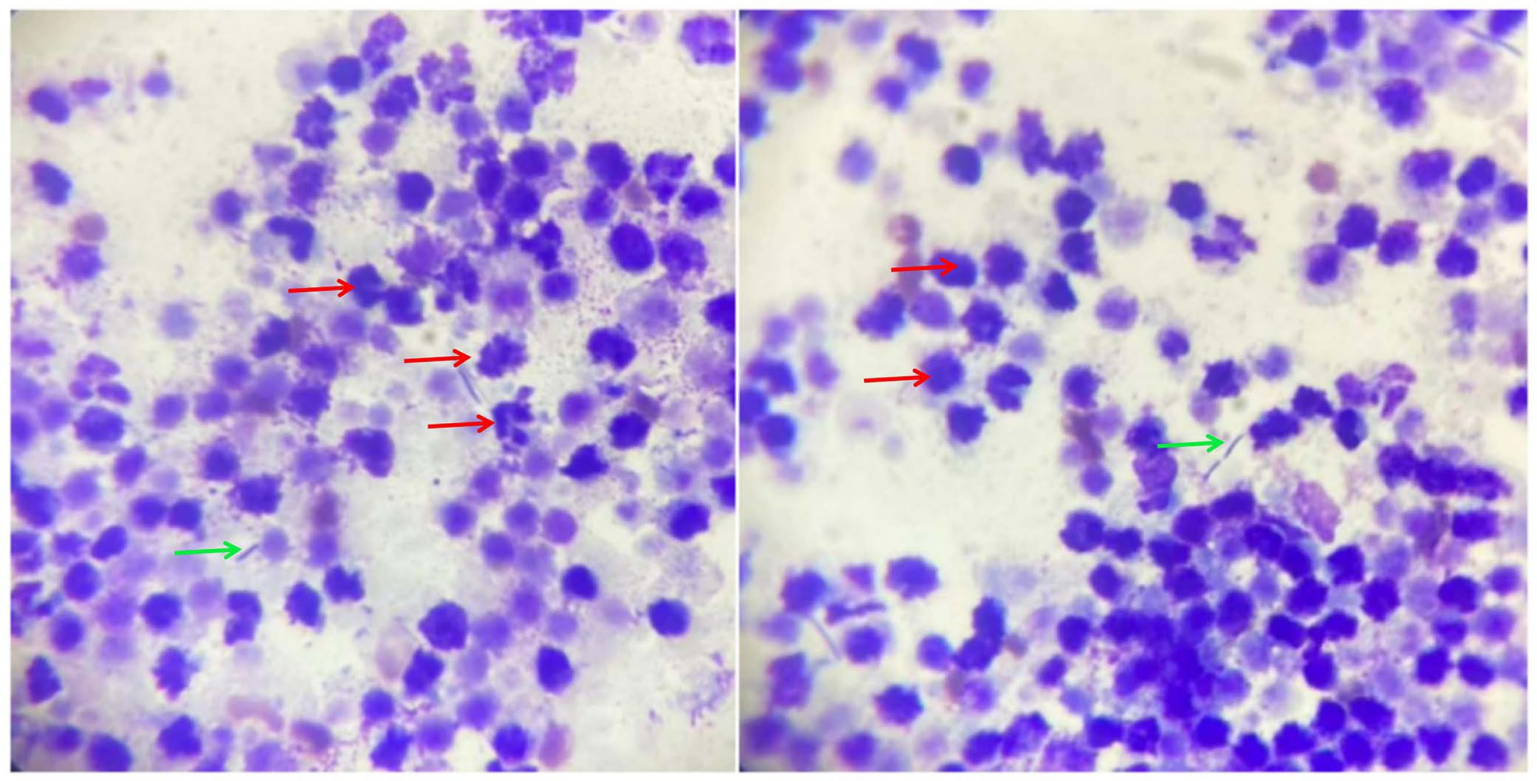
Cytological examination of prostate aspirates. The red arrows represent neutrophils and the green arrows represent rod-shaped bacteria.

Routine blood test results (hematology showed) showed that neutrophils, monocytes and c-reactive protein increased. Blood biochemistry showed increased alkaline phosphatase and decreased amylase. Routine urinalysis showed dark brown appearance with an increase in cast cells and red blood cells. Meanwhile, transitional epithelial cells were observed, suggesting a bacterial urinary tract infection. The biopsy fluid of the prostate contents was stained and viewed under a microscope, revealing a large number of degenerative neutrophils and rod-shaped bacteria (Figure 2).

### 2.4 Tumoral mass removal

According to the above examination results (e.g., dysuria, frequent urination and hematuria; elevated neutrophil count; swelling of the prostate; less uniform echo in the prostate region, no echo effect in parenchyma), a preliminary diagnosis of prostatic cyst was made. The owner was advised to take surgical treatment, but did not agree. Then antibiotic therapy was taken: amoxicillin clavulanate potassium (12.5 mg/kg, subcutaneous injection), enrofloxacin injection (10 mg/kg, subcutaneous injection), metronidazole (15 mg/kg, intravenous injection), and finasteride tablets (5 mg/time/day, oral). After 3 days, no significant improvement in symptoms was observed and the symptoms gradually worsened. After communication, the owner received omental packing for treatment.

**Figure 3:**
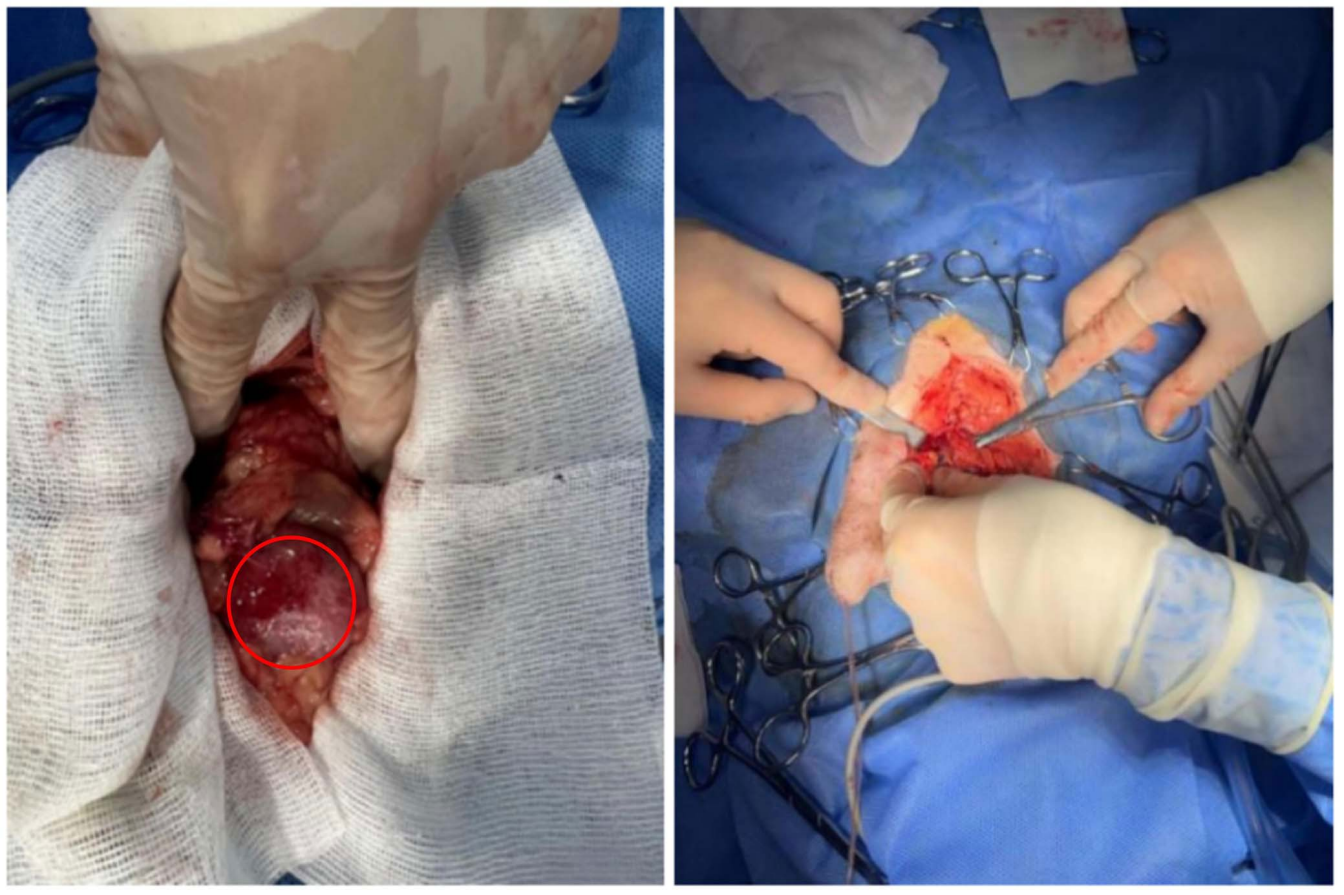
Enlarged prostate observed during surgery, as well as the sampling procedure. The red circle represents an enlarged prostate.

Propofol (5 mg/kg), teletamine (1 mg/kg), and zolazepam (1 mg/kg) were administered preoperatively. Anesthesia was induced with isoflurane (5%) and maintained with isoflurane (2%). Meloxicam (0.2 mg/kg) and butorphanol (0.2 mg/kg) were administered for analgesia. The surgical part was 2 cm lateral to the penis and 4 cm anteriorly from the anterior margin of the pubis. Once the enlarged prostate is located, sampling is performed (Figure 3). The abdominal cavity was washed with warm 0.9% sodium chloride injection. The greater omentum was introduced into the prostate tissue and fixed at the opening of the prostate using a nodular suture. The omentum of the other parts was wrapped and fixed with the prostate by intermittent nodular suture.

### 2.5 Histopathology

**Figure 4:**
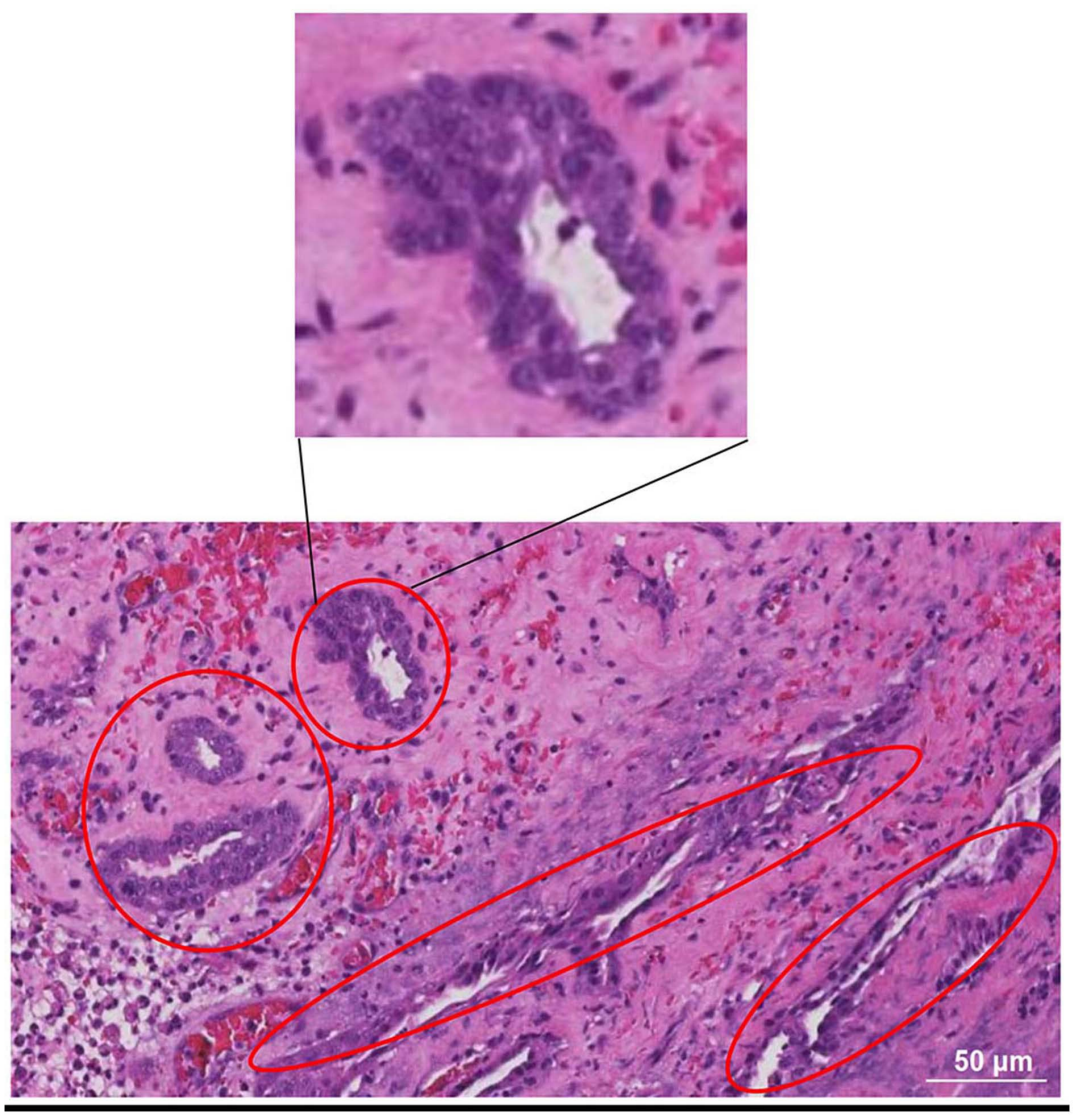
HE-stained photomicrograph of the prostate. It showed epithelial cell carcinoma infiltration, inferring that the dog suffering from prostate adenocarcinoma. The red oval boxes represent areas where cancer cells gather.

Prostate tissue fragment samples were obtained during the surgical procedures described above. They were immediately immersed in 10% formaldehyde fixative. At the end of fixation, after dehydration and wax immersion, paraffin tissue blocks were cut into 4 μm slices using a microtome. Finally, the samples were stained with hematoxylin and eosin and observed under a light microscope. The results showed epithelial cell carcinoma infiltration, inferring that the dog suffering from prostate adenocarcinoma (Figure 4).

### 2.6 Additional test

During the operation, after the contents of the prostate were extracted by puncture, bacterial culture identification and drug sensitivity test were performed. The extracts were cultured using blood plates + chocolate plates + MH plates, which showed *Enterobacter cloacae*. Drug susceptibility test results showed that: ampicillin, gentamicin, streptomycin, ciprofloxacin, ceftiofur, amikaci, tetracycline, chloramphenicol, marbofloxacin, and cefotaxime were the sensitive antibiotics.

### 2.7 Drug therapy

According to the results of pathogen identification and drug sensitivity, enrofloxacin (10 mg/kg/d, 1 week, oral), carprofen (45 mg/d, oral), mitoxantrone injection (2.5 mg/kg, intravenous injection, once every 3 weeks) were recommended. Follow-up and X-ray examination of the dog showed reduced prostate swelling, increased light transmission, and no significant space-occupying lesions in the skeletal system (Supplementary Figure S1). Therefore, the dog was in good condition and did not metastasize significantly after omental packing treatment.

## 3 Discussion

The dog presented with typical clinical signs of a prostatic abscess such as tenesmus and hematuria. Puncture fluid examination, however, was not diagnosed with prostate cancer. This is a dog who was initially diagnosed with a prostate abscess, which was confirmed by biopsy as prostate cancer, and treated with omental packing.

It is difficult to distinguish between prostate abscess and prostate cancer without biopsy (9, 10). CT examination and magnetic resonance imaging are a priority, but the high cost of the examination is unacceptable to many owners, thus limiting practical application. CT examination is of great application value when metastatic lesions are suspected (11). At present, invasive sampling for pathological tissue section examination is the most accurate basis for cancer diagnosis (12). In prostate cancer, some acinar structures can be observed in pathological tissue sections, while urothelial cells show tail shapes and sporadic cytoplasmic vacuoles with bright pink material; however, these features are not present in all cases (13). The tissue examined in this case showed epithelial cancer cell infiltration. Based on the clinical information and the location of the lesion, adenocarcinoma of the prostate is the most consistent diagnosis. Prostatic adenocarcinoma is not dependent on androgen (the dog has been castrated 6 years ago), and about 70–80% of cases will have aggressive move quickly, often can be transferred to the pelvis and lumbar lymph nodes, but are visible extensive transferred to the various organs, including bone (lumbar) and brain (14). Such lesions usually have a high mortality rate and short survival time, and are often associated with not being diagnosed until the advanced stage of the disease (15).

Total prostatectomy is the usual choice for the treatment of prostate cancer (16). However, some owners do not choose this method because it involves pubic incision, which is more destructive, has more complications, and costs more (9). The greater omentum has a strong ability of absorption, repair and anti-infection, and the application of omentum packing in the treatment of abscess cavity is effective (17–19). Meanwhile, omental packing is significantly better than antibiotic therapy. In this case, previous antibiotic therapy did not achieve good results. Because the affected dog is older, it is recommended that the owner not take the less destructive and safer omental packing. The dog did not develop complications such as urinary incontinence after surgery [the incidence of urinary incontinence after surgery has been reported to be 20–33% (20–22)], and the symptoms of hematuria were relieved 3 days after surgery. Therefore, it can be seen that omental packing is a method that can be considered for the treatment of prostate diseases. Although this is the report of treatment with this new method in dogs, this case report provides tentative evidence that the application of omental packing combined with antineoplastic drug injection is feasible, a large number of experimental samples are still needed for further observation.

## 4 Conclusion

In summary, the preferred treatment for prostate cancer is total prostatectomy with postoperative antineoplastic agents. For some cases diagnosed as prostatitis or prostate abscess in the early stage, but with prostate cancer in the later stage, there is a lack of similar treatment methods for omental packing in the early stage and drug anti-tumor treatment in the later stage. The case report described here provides a novel treatment modality for the treatment of prostate cancer.

## Data Availability

The original contributions presented in the study are included in the article/Supplementary material, further inquiries can be directed to the corresponding author.

## Supplementary material

SUPPLEMENTARY FIGURE S1 Follow-up and X-ray examination were performed after surgery. The blue circle represents the prostate region.
