# Supplementary figures and images for: Case report: A follow-up report of omental packing and drug therapy for canine prostate adenocarcinoma
Source: Front Vet Sci. 2024 Nov 12;11:1444684. doi: 10.3389/fvets.2024.1444684 (PMC11588718; doi:10.3389/fvets.2024.1444684)

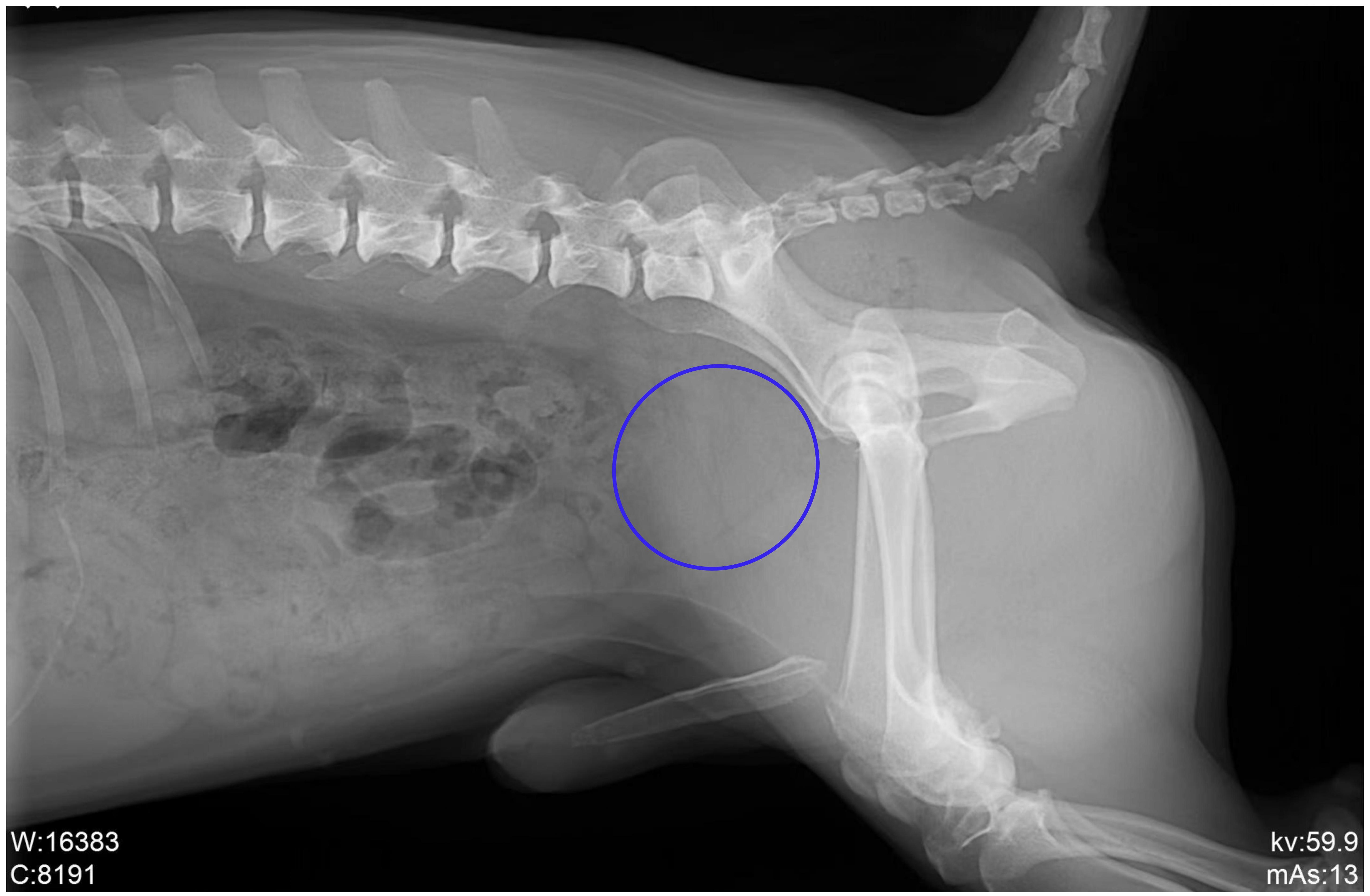

Supplement: SUPPLEMENTARY FIGURE S1 — Follow-up and X-ray examination were performed after surgery. The blue circle represents the prostate region. [file Image_1.jpeg]
